# *Citrus limon* peroxidase-assisted biocatalytic approach for biodegradation of reactive 1847 colfax blue P3R and 621 colfax blue R dyes

**DOI:** 10.1007/s00449-022-02802-z

**Published:** 2022-11-01

**Authors:** Arjumand Riaz, Umme Kalsoom, Haq Nawaz Bhatti, Teofil Jesionowski, Muhammad Bilal

**Affiliations:** 1 Department of Chemistry, Government College Women University Faisalabad, Faisalabad, Pakistan; 2 Department of Chemistry, University of Agriculture Faisalabad, Faisalabad, 38000 Pakistan; 3 Institute of Chemical Technology and Engineering, Faculty of Chemical Technology, Poznan University of Technology, Berdychowo 4, PL-60695 Poznan, Poland

**Keywords:** Environmental pollutants, Biocatalysis, *Citruslimon* peroxidase, 1847 Colafx Blue P3R, 621 Colafx Blue R, Dye decolorization

## Abstract

One of the big environmental problems in today’s world is dye-contaminated toxic waste. Peroxidase is known as highly efficient for the degradation of various pollutants, including dyes. Environmental contamination caused by the discharge of dyes into water bodies is an onerous challenge that poses both human and ecological hazards. In the current studies, biocatalysts used for enzyme decolorization (1847 Colafx Blue P3R and 621 Colafx Blue) are regarded as an eco-friendly method utilizing commonly available low-cost material lemon peels (*Citrus* *limon* peroxidase). Peroxidase was extracted in a phosphate buffer of pH 7.0 and partially purified by 20–80% ammonium sulfate precipitation technique from *Citrus* *limon* peels. The soluble enzyme was characterized in terms of kinetic and thermodynamic parameters. The values of Km and Vmax (23.16 and 204.08 μmol/ml/min) were determined, respectively. The enzyme showed maximum activity at pH 5.0 and a temperature of 55 °C. *Citrus* *limon* efficiently degraded 1847 Colafx Blue P3R and 621 Colafx Blue R dyes with maximum degradation of 83 and 99%, respectively, with an initial dye concentration of 200 ppm at pH 4 and 35 °C temperature within 5–10 min of incubation time. The effect of the redox mediator on the degradation process was examined. Results showed that the peroxidase HOBT system efficiently enhanced the degradation of dyes from water. Hence, *Citrus* *limon* peroxidase is an efficient biocatalyst for the treatment of effluents.

**Supplementary Information:**

The online version contains supplementary material available at 10.1007/s00449-022-02802-z.

## Introduction

The fast expansion of the industrial sector has resulted in a significant increase in water usage. The resulting wastewater characterized by high stains, intensive suspended solids, chemical oxygen demand (COD), and biochemical oxygen demand (BOD). It is, important to appropriately deal with these water bodies to secure life and the environment. Dyes are one of the significant problems in sewage streams as even in small quantities (1 ppm), they are highly visible. Colored water hurts living organisms. The dye-contaminated water may cause living beings illnesses, such as heart, liver, and nervous system damage [1].

Dyes are substances that absorb electromagnetic radiation in the visible spectrum, such as between 350 and 700 nm. Auxochromes and chromophores are both present in dye molecules. Chromophores are substances having delocalized electron systems, conjugated double bonds (–C=C–, –C=N, –C=O, –NO_2_, –N=N,) quinoid rings, and so on that transfer color to the dye molecule. The electrons withdrawing and donating groups –NH_2_, –COOH, –SO_3_H, –OH, are known as auxochromes. Auxochromes are used to change the overall energy of the molecular system’s electronic cloud to change the chromophore’s color [2]. Dyes are classified as azo, anthraquinone, heterocyclic, triphenylmethane, and polymeric dyes primarily based on the chemical structure of the chromophoric category, among which most textile dyes produced include the versatile azo and triphenylmethane dyes. These dyes must be treated to reduce their toxicity before disposal and discharge to major water bodies since they are mutagenic and poisonous and cannot be removed using conventional wastewater treatment methods remedies [3].

Dyes are commonly utilized in the food, cosmetic, leather, textile, paper, and plastic industries and then released into the atmosphere. The most extensively used reactive dyes pose a health risk, especially in powder form. Additionally, it found that dermatitis, rhinitis, and asthma symptoms are brought about by exposure to reactive dyes. There discharged into the environment due to the inefficacy of the dyeing process producing highly colored effluent and harmful effects [4].

Now dye treatment is immensely needed to prevent environmental contamination (Nouren et al., 2017). Inefficiencies in dyeing during textile processing result in considerable dyestuff loss (varying between 20 and 50% depending on the nature of the dyes) that is immediately discharged into water bodies as wastewater [2].

Several physical and chemical methods have been used to remove pollutants from wastewater, like electrochemical oxidation, coagulation–flocculation, membrane filtration, adsorption, etc. [5]. However, adsorption, precipitation, and ozonation are used to decolorize dye effluents have some limitations, including high costs, the emergence of hazardous by-products, intense power requirements, etc. [3]. Methods for detecting as well as remediation of dye contaminants are constantly being investigated. The development of several remediation methods to efficiently remove dye containments has made significant progress [6, 7, 8].

The peroxidase-assisted degradation process has been used for 2–3 decays to detoxify textile dyes. In 1999, the dye decolorizing peroxidase (A DyP) was found and isolated from a few sources, including the basidiomycetes *Bjerkandera Adusta* (fungus) and *Moringa* *oleifera*, etc. [9]. It is currently being employed by isolating from various sources with greater enzyme activity and thermal stability. In future, advanced materials can be used for the immobilization of enzymes as well as this process can be used industrially on a large scale.

The enzymatic methodology is enthusiastic about decolorizing the industrial and textile dyes from wastewater as an elective way to deal with physical, biological, and chemical treatments. In the presence of H_2_O_2_, peroxidase has been shown to oxidize a broad spectrum of organic substrates. Peroxidases (EC.1.11.1.x) are hydrogen peroxide (H_2_O_2_) decomposition enzymes that are involved in the oxidation of phenolic and non-phenolic substrates (RH) (Eq. 1).

1 $$2{\text{RH + H}}_{{2}} {\text{O}}_{{2}} \to {\text{2R + 2H}}_{{2}} {\text{O}}$$

They have been found in bacteria, fungi, algae, plants, and mammals worldwide. Plant peroxidases, which belong to the Class III peroxidase family, have a role in cell wall metabolism, lignification, suberization, reactive oxygen species (ROS) metabolism, auxin metabolism, fruit growth, and ripening, pathogen defense, etc. [10]. Peroxidases are thermally stable, highly effective in milder reaction conditions, and catalytically non-selective [11]. Peroxidases are associated with various physiological processes, including tissue healing and cross-connection of polysaccharide cell walls, tissue repair, pathogen resistance mechanisms, chloroplast and cytosol scavenging of H_2_O_2_, heavy metals detoxification, and reactive oxygen species during oxidative stress and cellular metabolic procedure, and biodegradation reactions. Moreover, it has also been stated that soluble and immobilized peroxidases can decolorize and eliminate synthetic dyes from industrial effluents and polluted water [12].

Plant-derived peroxidases, such as those from horseradish [13], soybean [14], Cucurbita pepo [15], turnip, and bitter gourd, are commonly employed to remove and degrade complex textile compounds [16], Turnip roots (Silva et al., 2012), *Nicotiana sylvestris* plants “woodland tobacco” (Gazaryan et al., 1995), Soybean hulls (Liu et al., 1999), Cauliflower buds (Koksal et al., 2008), *Raphanus sativus* (Bhatti et al., 2012), Tomato (Rathnamsamy et al., 2014), Wheat bran (Hamid et al., 2015), Rosemary leaves (Aghelan et al., 2015), bitter ground (Panadare and Virendra, 2017), Alfalfa Roots (Dubrovskaya et al., 2017), cauliflower stems (Ratanapongleka and Onsarn, 2018), Calotropis procera (Mafulul et al., 2018), cabbage leaves (Mehde, 2019), Fresh date palm (Khan et al., 2018) and Kinnow peels [17], *Prunus domestica*, plums (Enachi et al., 2018), (*Raphanus sativus L*.), Turkish black radish (Altinkaynak et al., 2017), fresh cut potato (Li et al., 2018) and Orange peels (Salgaonkar et al., 2019). Pakistan is a citrus-producing state, and the massive volume of waste peels produced is a good source of peroxidase [17, 18]. This study aims to learn more about the *Citrus* *limon* peroxidase-mediated decolorization of textile dyes. The impact of optimal pH, enzyme dose, H_2_O_2_ concentration, temperature, incubation time, dye amount, and redox mediator on textile dye degradation was investigated (Nouren et al., 2017).

## Materials and methods

### Chemicals

*Citrus* *limon* peels were collected from the local market, Jhang. Dyes were generously provided by True Colors, Faisalabad. The hydroxybenzotriazole (HOBT), redox reagent, ammonium sulfate, and all other chemicals and reagents were procured from Daejung Koria. The major part of research work was conducted in organic lab II, Government College Women University, Faisalabad.

### Extraction of Crude Enzyme

The crude extract was prepared by taking fresh lemon peels. *Citrus* *limon* peels at a ratio of 10 g leaves in 100 ml of buffer were homogenized with chilled (4 °C) phosphate buffer (0.1 M, pH 7.0.) in an electric grinder for 15 min with short intervals. Crude peroxidase extract was filtered through Whatman filter paper or cheesecloth to remove suspended particles. The extract was then filtered using vacuumed filtration assembly. The supernatant was stored at 4 °C.

### Ammonium sulfate precipitation and dialysis

The fine ground ammonium sulfate (NH_4_)_2_ SO_4_ was used to precipitate peroxidase in an ice bath. The powder was weighed and slowly added by continuous stirring to extract. Adding eighty percent (w/v) of ammonium sulfate, the solution was subjected to salt fractionation, kept overnight at 4 °C, and then centrifuged at 10,000 rpm using a refrigerated high-speed centrifuge machine for 15 min. In a small quantity of 100 mM sodium acetate buffer (pH 7.0), precipitates were dissolved and dialyzed against the pH 7 buffer (5 times) [19].

### Enzyme characterization and assay

Peroxidase activity is evaluated calorimetrically using a spectrophotometer (Cecil 7200) following tetraguaiacol formation at *λ*_max_ = 470 nm. The quantity of enzyme that catalyzes 1 mol of guaiacol in 1 min is defined as one unit of peroxidase activity [11]. The optimum pH for peroxidase activity was achieved by measuring the enzyme's activity using buffers in the pH range of 2–10: glycine HCL (pH 2–4), acetate (pH 5–6), phosphate (pH 7–8) and tris–HCl (pH 9–10) [9]. The *K*_m_ and *V*_max_ for guaiacol for *Citrus* *limon* peroxidase were examined. Various concentrations of guaiacol (C_7_H_8_O_2_) 6–30 mM were used to investigate the effect of guaiacol on peroxidase activity while the amount of H_2_O_2_ was kept constant [9]. The optimal temperature was evaluated by measuring enzyme activity at various temperatures (25–80 °C). The enzymes were placed at high temperatures; the kinetics, and thermodynamics parameters were assessed for thermal denaturation of *Citrus* *limon* peroxidases, such as Gibbs free energy change (ΔG*), enthalpy change (ΔH*), and entropy change (ΔS*) according to the following equations (Eqs. 2, 3, 4) [20].

2 $$\Delta H\left( {\frac{{{\text{KJ}}}}{{{\text{mole}}}}} \right) = Ea - RT{ }$$

3 $$\Delta G\left( {\frac{{{\text{KJ}}}}{{{\text{mole}}}}} \right) = - RT{\text{In}}(\mathop K\nolimits_{{\text{d}}} \times h/K_{{\text{b}}} )$$

4 $$\Delta S\left( {\frac{{{\text{KJ}}}}{{{\text{mole}}}}} \right) = \Delta G - \Delta H/T\left( K \right)$$

Here, *Ea* is the activation energy, *R* is constant (8.314 J/mole), and *T* is the Temperature in Kelvin. *K*_b_ is the Boltzmann constant (1.38 × 10^−23^JK^−1^) and *h* is Planck’s constant (6.62 × 10^–34^ Js).

### Decolorization of dyes with Citrus Lemon Peroxidase (CLP)

Each of the dyes was incubated for 1 h at 35 °C with the enzyme in the presence of a buffer of (pH 5.0), H_2_O_2_ in a water bath shaker. After that, the sample was centrifuged (at 10,000 rpm for 10 min), and the absorbance was measured. Equation 5 was used to compute the % decolorization [21].

5 $${\text{Decolorization }}\left( \% \right) \, = \, \left[ {\left( {A_{{\text{i}}} - A_{{\text{f}}} /A_{{\text{i}}} } \right)} \right] \, \times {1}00$$

### Optimization of peroxidase activity for Dye Decolorization

The process of dye decolorization with *Citrus* *limon* peroxides was used in different experiments to determine the varying effect of reaction variables within ideal reaction mixtures. The first pH was optimized, ranging from pH (2–10). Similar experiments were conducted to optimize temperature (25 °C–95 °C), enzyme dose (0.05–0.2), and H_2_O_2_ concentration (0.05–0.2 mM). The incubation time (5–60 min) was calculated [21]. All the studies were carried out in test tubes with a shaking water bath. The impact of dye dose on decolorization was examined by changing the primary amount of dyes ranging from 100 to 400 ppm in the presence of 0.1 mL of H_2_O_2_ while keeping other parameters constant in pH 5 [22]. Triplicates of each assay and decolorization experiment were run, and standard deviations were computed.

## Results and discussion

### Partial purification and extraction of peroxidase

Activity for the crude enzyme was recorded as 151.89 U/mg for lemon peroxidase. The crude enzyme was then purified using 80% ammonium sulfate (NH_4_)_2_ SO_4_ and centrifuged for 15 min at 4 °C and 10,000 rpm. Then excess ammonium sulfate was removed through dialysis. Enzyme activity increased to 304.52 U/mg [11].

### Characterization of Citrus limon Peroxidase

Different kinetic or process parameters were optimized for peroxidase characterization.

#### pH Effect and pH Stability

**Fig. 1:**
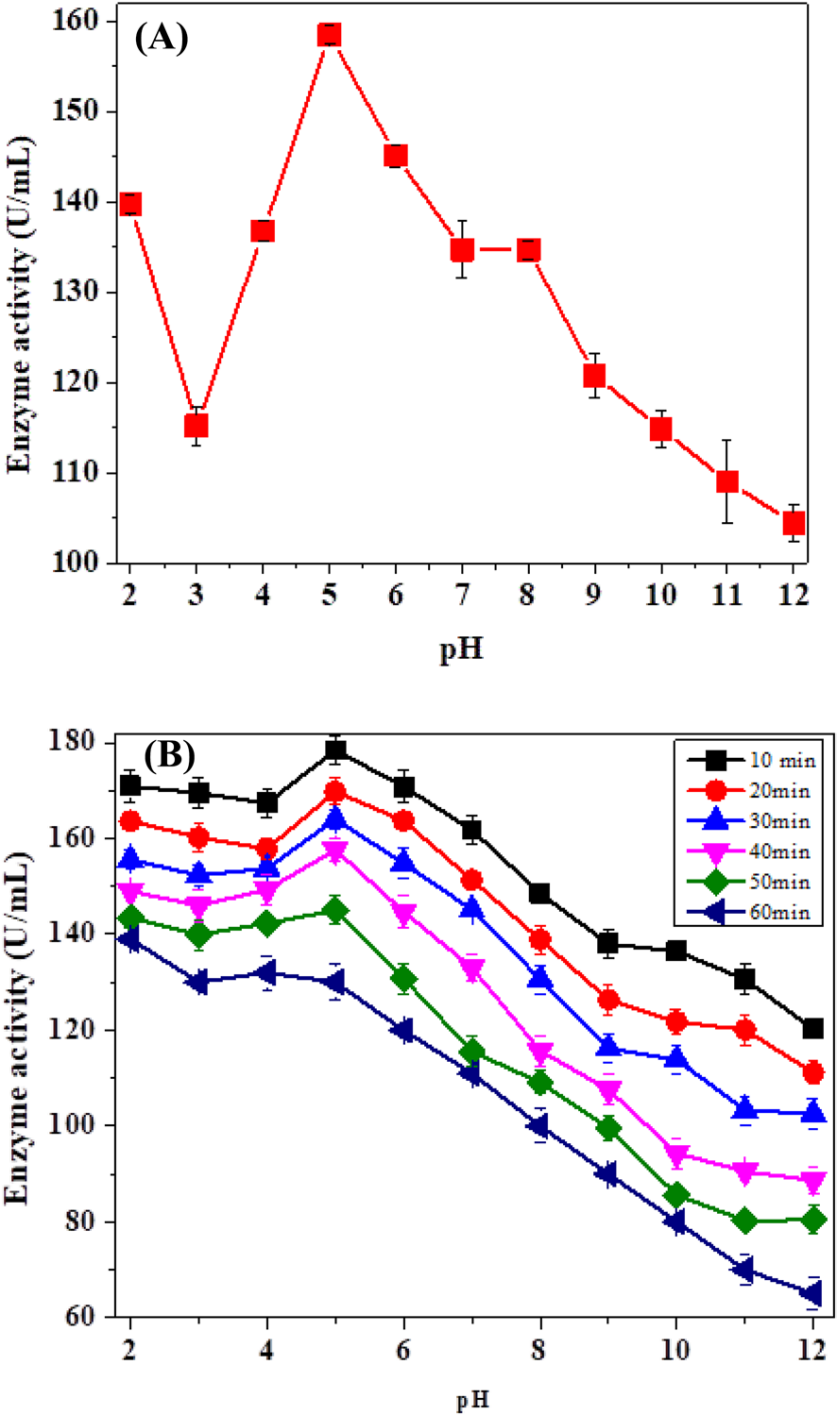
**A** Effect of pH on the enzyme activity and **B** pH stability profile

For optimal pH, the behavior of *Citrus limon* peroxidase (CLP) was shown through graph Fig. 1a utilizing pH buffers raging (2–12). At pH 5, CLP activity was observed at its peak. Subsequently, there was a gradual increase from pH 3 to 5 and a decrease in activity as the pH increased to 6–12. A similar trend was observed in a purified enzyme from a medicinal tree *Azadirachta indica* peroxidase, which exhibited maximal activity at pH 5 [10]. To assess the pH stability, *Citrus* *limon* peroxidase was incubated at different temperatures (35, 40, 45, 50, 55, and 60 °C) for different time intervals using buffers of varying pH 2–12 and kept overnight at 4 °C, that pH stability was assayed spectrophotometrically. In Fig. 1b, pH stability trend was shown for the enzyme. The pH stability experiment revealed that the partly purified peroxide was stable throughout a wide pH range (3–12).

#### Temperature effect and thermal stability

**Fig. 2:**
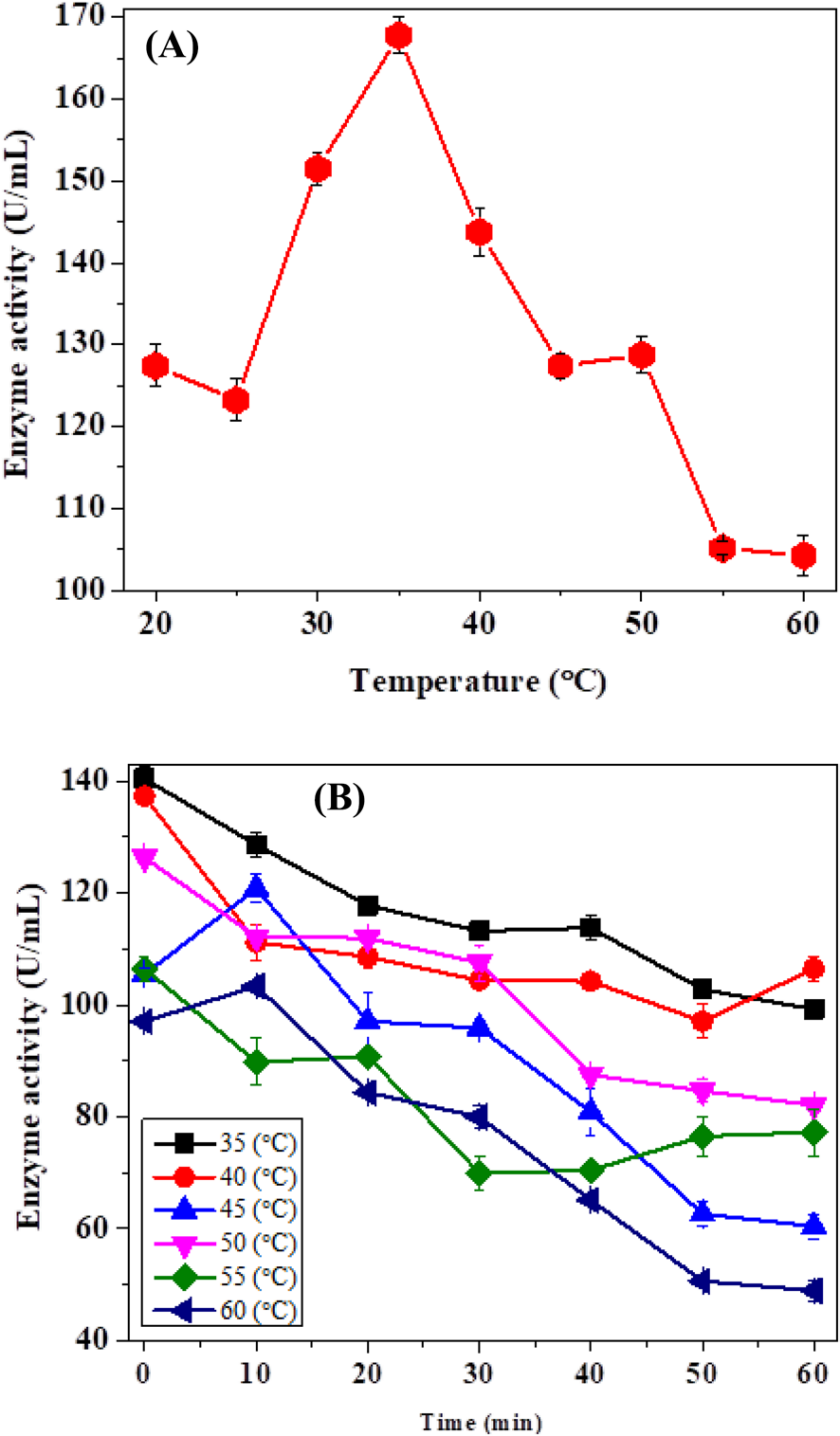
**A** Effect of temperature on the enzyme activity and **B** temperature stability profile

Biocatalysts are sensitive and can be denatured in severe physical and chemical conditions. Elevated temperature is the obvious factor that leads to the denaturation of enzymes by changing the structural conformation of protein molecules. It is, therefore, necessary to determine the thermal stability of enzymes at high temperatures for industrial applications. The temperature activity profile and thermal stability were shown by graph for CLP in Fig. 2a, b. Maximum temperature for enzyme efficiency was obtained by testing enzyme’s activity at multiple temperatures at pH 5.0 from 20 to 60 °C. The maximum activity was exhibited at 35 °C by CLP. For the thermal stability of POD, the 3 mL of peroxidase in a test tube was taken and incubated at temperatures 35, 40, 45, 50, 55, and 60 °C for time gaps (0, 10, 20, 30, 40, 50 and 60 min). Then enzyme was cooled in an ice bath and put at 4 °C overnight, and the enzyme activity was measured spectrophotometrically. Enzyme retained its activity at 60 °C showing a similar trend to that of peroxidase used by Darwesh (2019), and cabbage waste peroxidase [23].

#### Substrate concentration

**Fig. 3:**
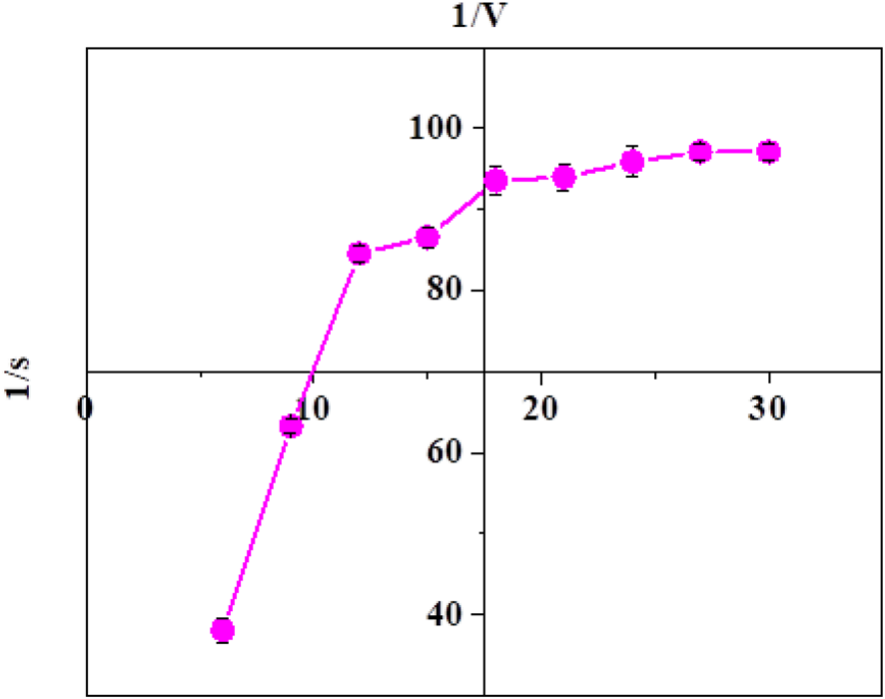
Guaiacol concentration effect on the activity of *Citrus limon* peroxidase

Peroxidase activity was measured at different concentrations (6–30 mM) of the substrate guaiacol to investigate the substrate affinity and kinetics of *Citrus* *limon* peroxidase. The effect of guaiacol on CLP can be seen in Fig. 3. The impact of substrate concentration and kinetic parameters, and substrate-specific (*K*_m_ and *V*_max_) values for guaiacol were found. The *K*_m_ and *V*_max_ were 23.16 and 204.08 μmol/ml/min.

### Thermodynamic parameters

Enthalpy, entropy, and free energy are important thermodynamic characteristics that determine protein unfolding during thermal inactivation. The system’s enthalpy and entropy represent the fundamental heat needed by the enzyme for its transformation from its initial state to transition [24]. These two factors represent the total number of non-covalent bonds, primarily hydrogen bonds broken in the overall changes brought by thermal inactivation (Zeyadi et al., 2020). The results of the thermal inactivation of the enzyme have been presented in Table 1. With temperature rise, the findings for enthalpy change (Δ*H*°) exhibited a decreasing pattern, showing a less energy demand for thermal denaturation at elevated temperatures. Enzyme’s stable behavior has been demonstrated by the results, which reveal that at 35 °C *Citrus* *limon* peroxidase had a half-life of 330 min, which decreased with increasing temperature until it reached 173 min at 60 °C. The free energy (Δ*G*º) observed was 104.047 kJ/mol at 35 °C. At higher temperatures, an increase in free energy value was observed 162.45 kJ/mol at 60 °C, illustrating *Citrus* *limon* peroxidase resistance to higher temperatures. The Δ*S*° values also showed a continuous slight decrease at (35–60 °C) with temperature increase [11].

**Table 1:** Thermodynamic parameters for *Citrus* *limon* peroxidase

Temp ^o^C	Temp (K)	*K*_d_ (1/min)	*K*_d_/s	*t*_1/2_	Δ*H*^o^ (kJ/mole)	Δ*G*^o^ (kJ/mole)	Δ*S*^o^ (kJ/mole)
35	308	0.0021	0.126	330	19.31425	104.047	18.97644
40	313	0.0025	0.15	277.2	19.27268	118.42	18.89434
45	318	0.0031	0.186	223.5484	19.23111	136.23	18.80272
50	323	0.0036	0.216	192.5	19.18954	149.65	18.72623
55	328	0.0036	0.216	192.5	19.14797	151.96	18.68468
60	333	0.004	0.24	173.25	19.1064	162.45	18.61857

### Dye Decolorization by *Citrus limon* Peroxidase

Dye deterioration experiments were performed by adding hydrogen peroxides to a buffer (pH 5) consisting of a dye solution containing *Citrus* *limon* peroxidase. Purified peroxidase was evaluated for degradation of various industrial dyes like Disperse (Turquoise 8080, Golden GG 8077, Blue 2BUV 8079, Turquoise 8082) and many others involving Reactive MCJ Prutug color, Reactive by Funcial, Finacial Salfuox, Direct azo-free, and non-azo-free dyes. First, the stock solution was made by taking 0.1 g/100 ml dye. Then the *λ*_max_ was determined using a UV–Visible spectrophotometer that was used as a standard to compare or calculate the percentage of decolorization of dyes using the peroxidase. The dye solution was incubated for 1 h at 35 °C with the enzyme (CLP) with reaction parameters like buffer (pH 5.0), and H_2_O_2_ (1 mM) in a water bath shaker. Some dyes exhibited decolorization with *Citrus limon*. The dyes that showed maximum decolorization with *C. limon* peroxidase, belong to the reactive by functional class. Dye 1847 Colafx Blue P3R and 621 Colafx Blue R demonstrated considerable degradation. Approximately 61 and 64% of dye removal were obtained in 1 h of incubation. The color change before and after decolorization using *Citrus* *limon* peroxidase was shown in Figures S1 and S2.

#### Parameters optimization for optimal dye degradation percentage

**Fig. 4:**
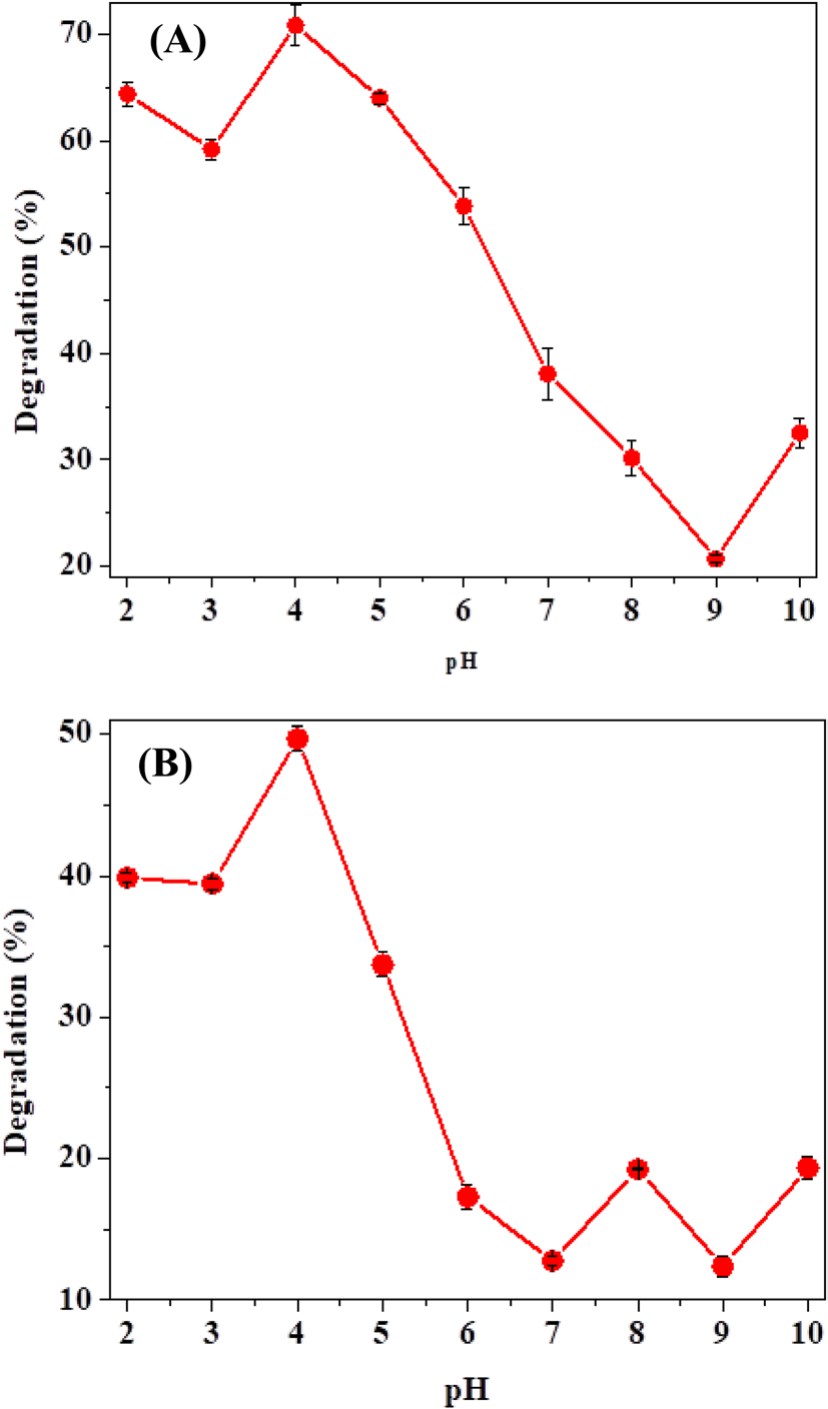
**A** Effect of pH on 1847 Colafx Blue P3R and **B** 621 Colafx Blue R dyes degradation

The decolorization rate at each pH (2–9) for 1847 Colafx Blue P3R was (64, 59, 70.55, 63.99, 55, 38, 30, 20.66 and 32.5%) and for 621 Colafx Blue R (40, 39.4, 50, 34, 16, 12.7, 18, 13 and 19.5%) respectively. The results are shown in (Fig. 4a, b). At higher pH, dyes demonstrated less degradation. Optimum decolorization (70.3 and 50%) was examined for 1847 Colafx Blue P3R and 621 Colafx Blue R, respectively, by *Citrus* *limon* peroxidase at pH 4 similar to *Ziziphus mauritiana* leaves peroxidase [25], soybean peroxidase [26] in contrast to Ginger peroxidase [27], Luffa Peroxidase [28], HRP that showed approximately 19% dye degradation at pH 4 [1, 29].

**Fig. 5:**
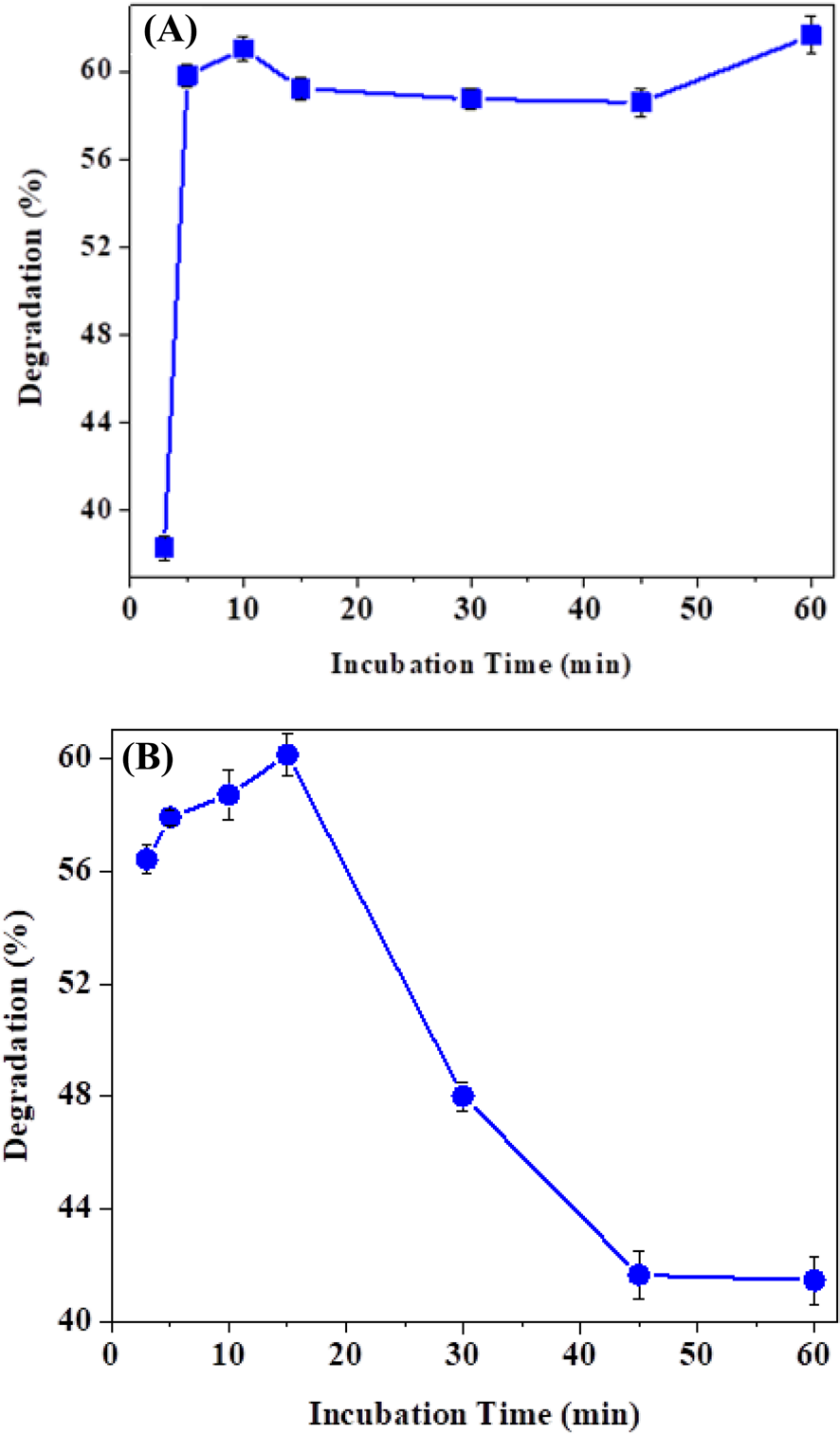
**A** Effect of incubation time on 1847 Colafx Blue P3R and **B** 621 Colafx Blue R dyes degradation

The contact duration between the catalyst and the substrate is a key factor in dye degradation. So, investigations were carried out to determine the optimal contact time required for *Citrus* *limon* peroxidase to decolorize 1847 Colafx Blue P3R and 621 Colafx Blue R. Time of incubation (5–60 min) was observed [21]. The incubation effect on both selected dyes is demonstrated in Fig. 5a, b. The dye decolorization % was determined, and the ideal incubation period was used for the next experiments. By evaluating the effect of reaction time, the maximum degradation was obtained up to 5–10 min, demonstrating the excellent efficiency of *Citrus* *limon* peroxidase for dye degradation.

**Fig. 6:**
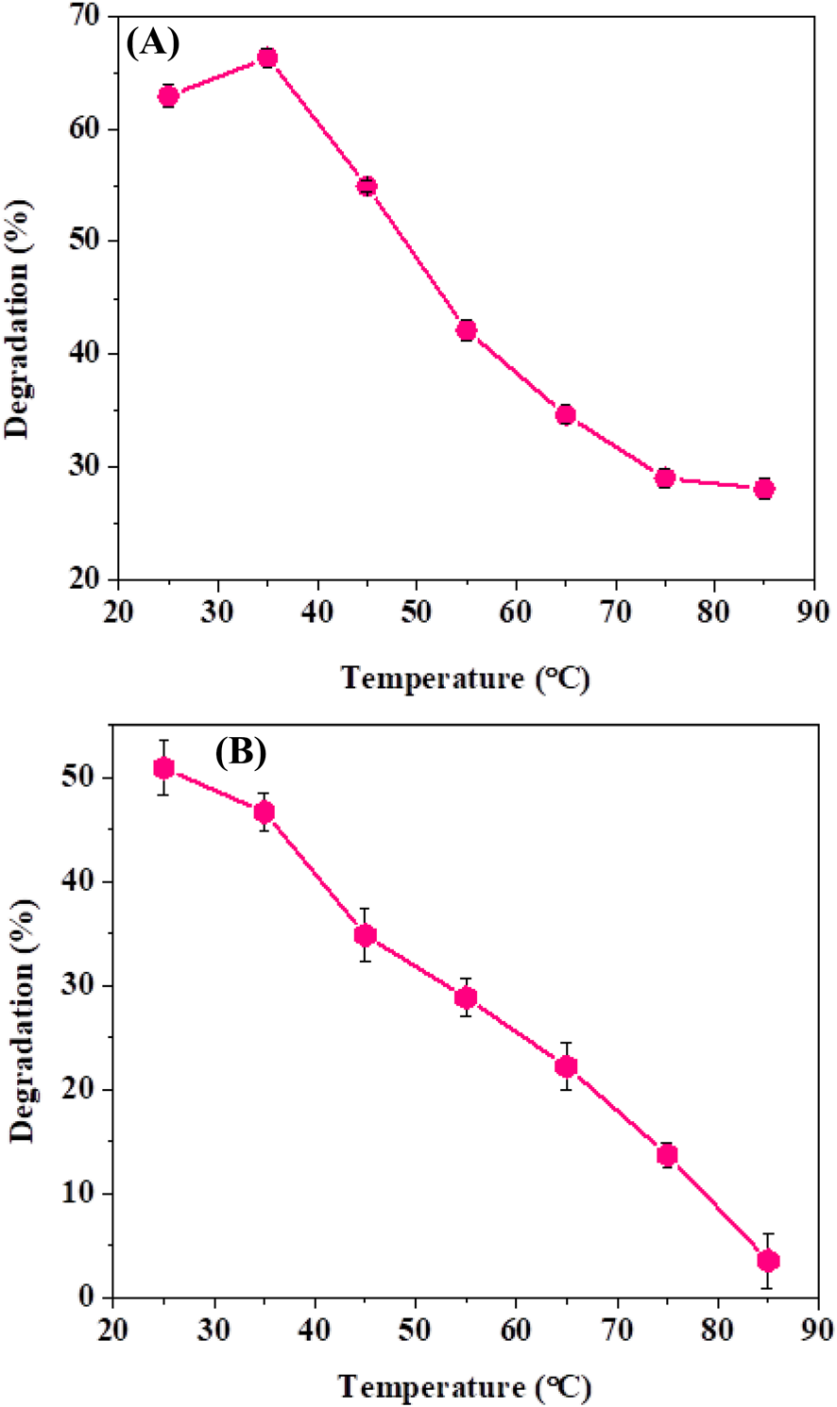
**A** Effect of temperature on 1847 Colafx Blue P3R and **B** 621 Colafx Blue R dyes decolorization

One of the most critical factors influencing enzyme function is temperature. The enzyme activity increases as the temperature rises as additional energy are readily accessible to increase the rate of reaction unless an ideal temperature value is reached at which the enzyme indicates higher activity, after which the enzyme activity begins to decline due to denaturation of the enzyme with temperature rise. Both dyes’ maximum deterioration showed in Fig. 6a, b up to 80% at 35 °C. Compared to soybean peroxidase which showed 45% degradation at 35 °C (Mandujano et al., 2018), Luffa Peroxidase causes 55% degradation [28]. Dyes were treated individually with 0.14 mL of peroxidase in the presence of 0.1 M H_2_O_2_ at the maximum reaction (4 pH) with an incubation period of 5–10 min in a water bath shaker at a temperature range (25–95 °C).

**Fig. 7:**
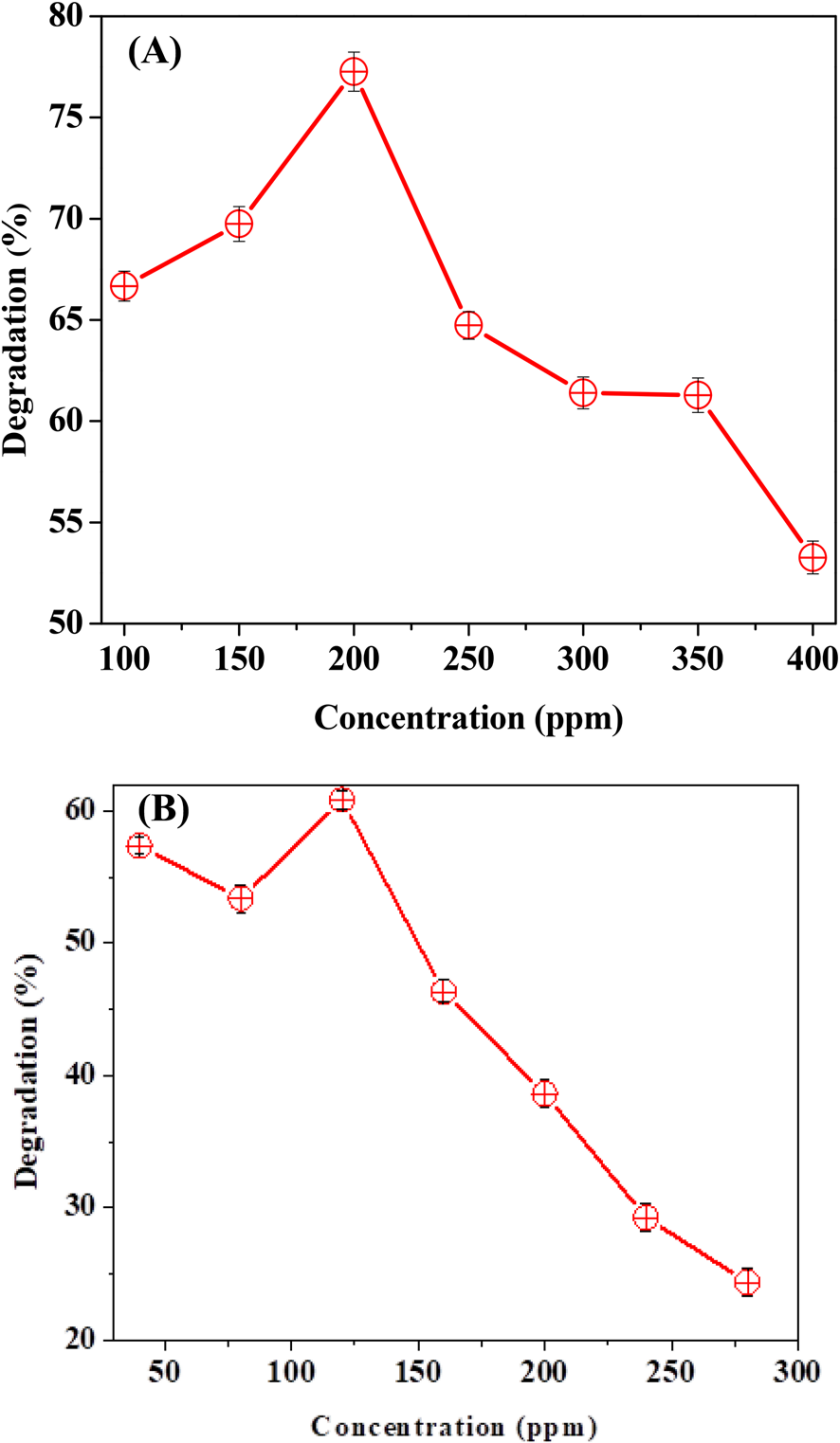
**A** Effect of dye concentration on 1847 Colafx Blue P3R and **B** 621 Colafx Blue R dyes degradation

The concentration of the dye that functions as a substrate in enzyme-catalyzed dye degradation processes had a direct impact on the reaction. The dye concentration was slowly raised while the enzyme quantity remained constant. The rate of reaction will continue to increase until a saturation point is achieved, at which further increase in the dye concentration will not affect the rate of reaction. The impact of dye dose on decolorization was examined by changing the primary amount of dyes ranging (from 100 to 400 ppm for 1847 Colafx Blue P3R and 50–300 ppm for 621 Colafx Blue R) in the presence of 0.14 mL enzyme, 0.1 mL of H_2_O_2_ and other parameters kept constant. The optimum color removal (77.26%) occurred up to 200 ppm dye solution for 1847 Colafx Blue P3R and 621 Colafx Blue R (61.7%) up to 120 ppm solution as shown in Fig. 7a, b.

**Fig. 8:**
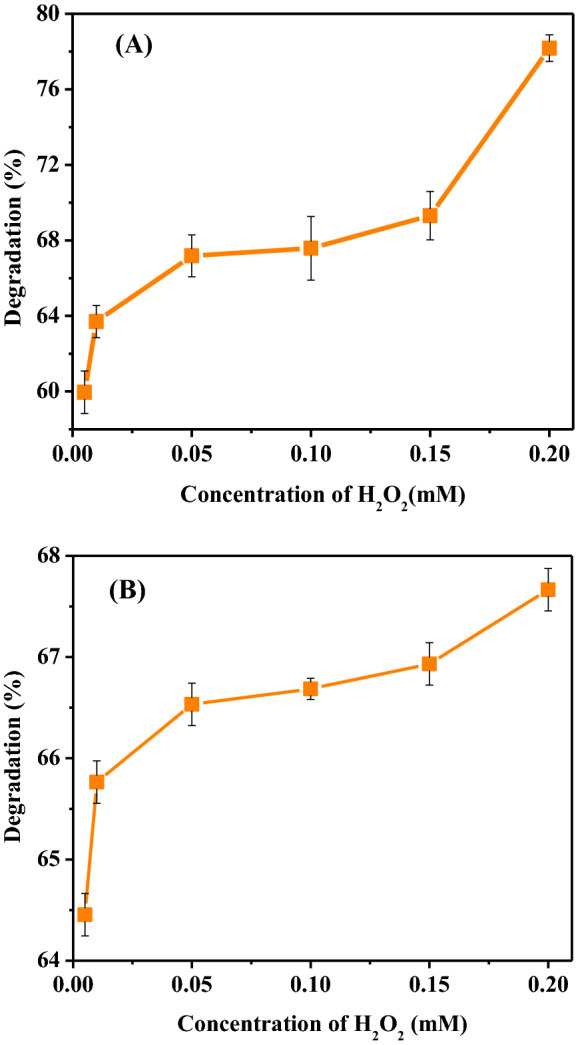
**A** Effect of H_2_O_2_ concentration on 1847 Colafx Blue P3R and **B** 621 Colafx Blue R dyes degradation

As a co-substrate, hydrogen peroxide participates in the catalytic reaction of the peroxidase by oxidation of the native enzyme to generate an intermediate, which then accepts one electron from the dye molecule. The influence of H_2_O_2_ concentration on the degradation percentage of dye was demonstrated in Fig. 8a, b. To determine the ideal concentration of H_2_O_2_ in the degradation of 1847 Blue P3R and 621 Blue R dyes, reactions were carried out by changing H_2_O_2_ concentration from 0.05 to 0.2 mM with optimal conditions of the incubation period, enzyme volume, pH, temperature, and concentration of dye. Decolorization efficiency of both dyes increased with higher H_2_O_2_ concentration to 80 and 63%. *Citrus* *limon* peroxidase showed better decolorization than Soybean waste peroxidase [22]. The same trend was shown by Soybean Peroxidase and Luffa Peroxidase [28].

**Fig. 9:**
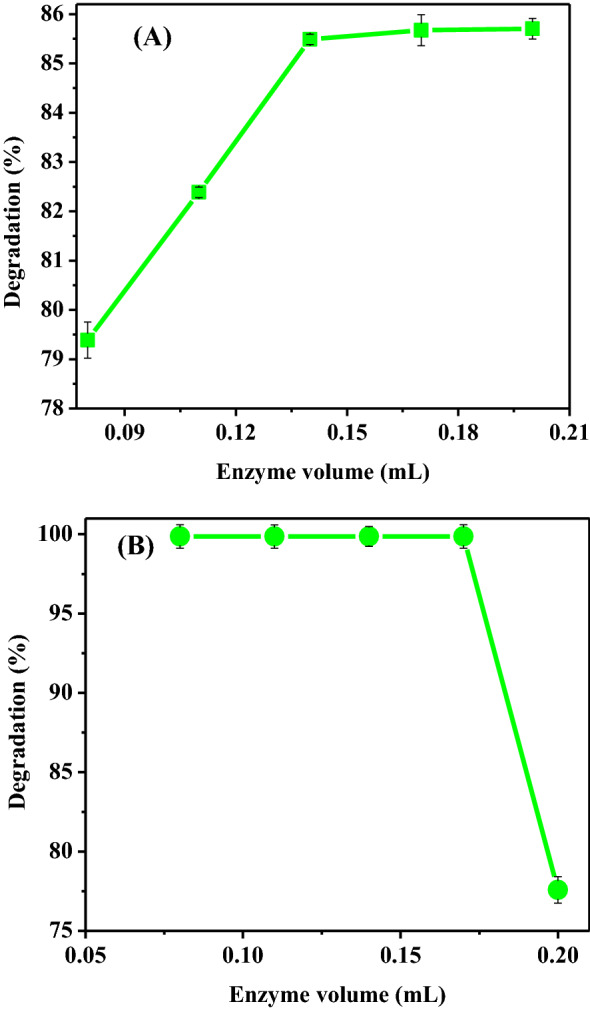
**A** Effect of enzyme dosage on 1847 Colafx Blue P3R and **B** 621 Colafx Blue R dyes degradation

As the enzyme dosage increased, the rate of enzyme-catalyzed processes was raised. This linear connection indicates that the reaction rate and enzyme dosage were proportionate. As a result, dye removal was influenced by the amount of enzyme added to a reaction mixture as well as the contact duration. Because enzymes have a finite life span as catalysts, there is always an optimal ratio between enzyme dosage and substrate to ensure maximal elimination. The effect of *Citrus* *limon* peroxidase dose on dye degradation was shown in Fig. 9a, b. The varying volume of peroxidase allowed maximum degradation of both dyes to be observed. Dyes 1847 Colafx Blue P3R and 621 Colafx Blue R were treated with varying volumes of an enzyme using 0.1 mL of 0.1 M H_2_O_2_ and with other optimal parameters like pH, temperature, and incubation time. Maximum degradation was observed at about 83.3% (1847 Colafx Blue P3R) for 0.14 mL of enzyme and 99% (621 Colafx Blue R) at almost all taken doses of the enzyme, whereas soybean waste and Luffa Peroxidase caused 85 and 75% decolorization using greater volume (0.5 and 3 mL), respectively.

#### Absorption Spectra of Dyes

**Fig. 10:**
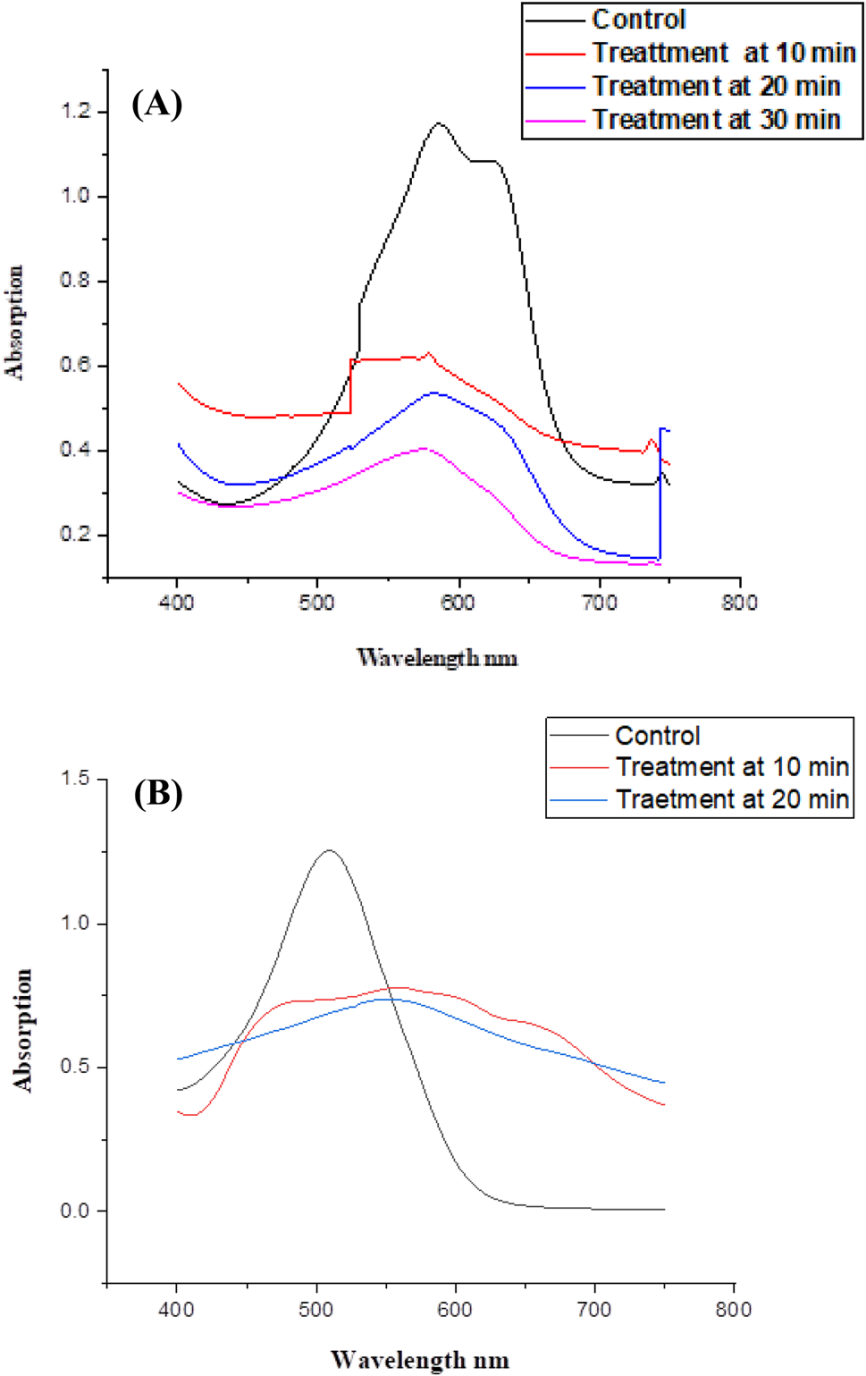
**A** Absorption spectrum of 1847 Colafx Blue P3R, and **B** 621 Colafx Blue R

The UV–Vis analysis of 1847 Colafx Blue P3R and 621 Colafx Blue R decolorization. The decolourization rate was measured in terms of the intensity of the absorption peak in the visible area ranging from 400 to 800. Absorption spectra of dyes treated by *Citrus* *limon* peroxidase showed in Fig. 10 using a UV–Visible spectrophotometer at time intervals of 0, 10 up to 30 min, etc. The behavior of dyes at standard conditions by *Citrus* *limon* Peroxidase at different time intervals was demonstrated.

## Conclusion

Due to its toxicity, carcinogenicity, and impact on aquatic life, dye wastewater from the textile and dyestuff industries must be managed. Because of the various and complicated molecular structures of dyes, dye wastewater is difficult to treat using conventional biological and physicochemical techniques. Therefore, it is crucial to investigate innovative treatment technologies for their remedies. This study showed that wastewater dyes might be efficiently treated using enzymes generated from cheap and accessible plant sources. *Citrus limon* peroxidase was shown to be a possible biocatalyst in this study for the decolorization of textile colors. The use of *Citrus limon* peroxidase for dye degradation is a cost-effective method due to its high enzyme activity, thermal stability, and requirement of less incubation time. Moreover, the inclusion of the redox mediator increased the decolorization percentage and reduced the incubation period. Maximum degradation achieved were 83 and 99%, for 1847 Colafx Blue P3R and 621 Colafx Blue R dyes respectively, under standard conditions. These properties and being readily accessible, cost-effective, and environmentally favorable biocatalysts make *Citrus* *limon* peroxidase a potential candidate to be used on an industrial scale for effluent treatments.

## Supplementary Information

Below is the link to the electronic supplementary material.

Supplementary file1 (DOCX 212 KB)
