# Supplementary material for: Citrus limon peroxidase-assisted biocatalytic approach for biodegradation of reactive 1847 colfax blue P3R and 621 colfax blue R dyes
Source: Bioprocess Biosyst Eng. 2022 Nov 1;46(3):443–52. doi: 10.1007/s00449-022-02802-z (PMC9950157; doi:10.1007/s00449-022-02802-z)
Supplement: Supplementary file 1 — Supplementary file1 (DOCX 212 KB) [file 449_2022_2802_MOESM1_ESM.docx]

**Citrus Limon Peroxidase-Assisted Biocatalytic Approach for Biodegradation of Reactive 1847 Colfax Blue P3R and 621 Colfax Blue R Dye Pollutants**

Arjumand Riaz ^1^, Umme Kalsoom ^1,^*, Haq Nawaz Bhatti ^2^, Teofil Jesionowski ^3^,

Muhammad Bilal ^3,^*

^1^ Department of Chemistry, Government College Women University Faisalabad, Pakistan.

^2^ Department of Chemistry, University of Agriculture Faisalabad-38000, Pakistan.

^3^ Institute of Chemical Technology and Engineering, Faculty of Chemical Technology, Poznan University of Technology, Berdychowo 4, PL-60695 Poznan, Poland.

*Corresponding author's emails: [dr.ummekalsoom@gcwuf.edu.pk](mailto:dr.ummekalsoom@gcwuf.edu.pk) (Umme Kalsoom); [muhammad.bilal@put.poznan.pl](mailto:muhammad.bilal@put.poznan.pl) (M. Bilal).


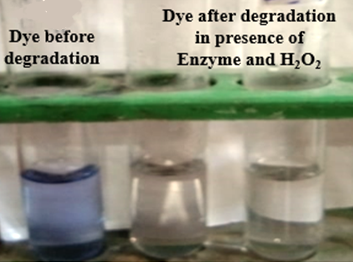


**Fig. S1** Dye 1847 Colfax Blue P3R degradation in presence of CLP


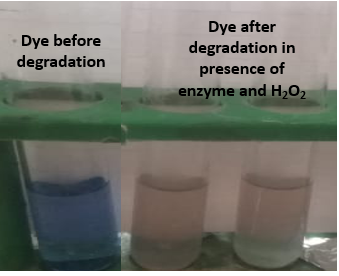


**Fig. S2** Dye 621 Colfax Blue R degradation in presence of CLP

Test tube **A** contains control involving (Dye + Buffer, pH 5.0)

Test Tube **B** involve (Dye + Buffer, pH 5 + Hydrogen peroxide + Enzyme)

Since two dyes 1847 Colfax Blue P3R and 621 Colfax Blue R display the maximum degradation with CLP, these two dyes further characterized using various factors like pH effect, Incubation time, temp effect, Substrate effect etc.
